# How to Restore Oxidative Balance That Was Disrupted by SARS-CoV-2 Infection

**DOI:** 10.3390/ijms23126377

**Published:** 2022-06-07

**Authors:** Kajetan Kiełbowski, Mariola Herian, Andrzej Pawlik

**Affiliations:** Department of Physiology, Pomeranian Medical University in Szczecin, 70-111 Szczecin, Poland; kajetan.kielbowski@onet.pl (K.K.); mariola.herian@gmail.com (M.H.)

**Keywords:** SARS-CoV-2, COVID-19, antioxidants, oxidative stress, cytokine storm

## Abstract

Coronavirus 2019 disease (COVID-19) is caused by different variants of severe acute respiratory syndrome coronavirus 2 (SARS-CoV-2) which emerged in December of 2019. COVID-19 pathogenesis is complex and involves a dysregulated renin angiotensin system. Severe courses of the disease are associated with a dysregulated immunological response known as cytokine storm. Many scientists have demonstrated that SARS-CoV-2 impacts oxidative homeostasis and stimulates the production of reactive oxygen species (ROS). In addition, the virus inhibits glutathione (GSH) and nuclear factor erythroid 2-related factor 2 (NRF2)—a major antioxidant which induces expression of protective proteins and prevents ROS damage. Furthermore, the virus stimulates NOD-, LRR- and pyrin domain-containing protein 3 (NLRP3) inflammasomes which play a significant role in inducing a cytokine storm. A variety of agents with antioxidant properties have shown beneficial effects in experimental and clinical studies of COVID-19. This review aims to present mechanisms of oxidative stress induced by SARS-CoV-2 and to discuss whether antioxidative drugs can counteract detrimental outcomes of a cytokine storm.

## 1. Introduction

Since the first infection of severe acute respiratory syndrome coronavirus 2 (SARS-CoV-2) in 2019, the disease has spread, causing a pandemic [1]. Coronaviruses are known to cause mild infections of the upper respiratory tract. However, there have been three viruses with the ability to replicate in the cells of the lower respiratory tract: Middle East respiratory syndrome (MERS), SARS-CoV and SARS-CoV-2. Infections with these pathogens may lead to life-threatening hypoxia [2]. Clinical presentation of COVID-19 infection is heterogenous and may include a variety of cold symptoms (e.g., fatigue, fever, cough) as well as symptoms from other systems: gastrointestinal, neurological or cardiovascular [3]. It is considered that a severe course of infection is associated with hyperinflammation and a dysregulated response to the pathogen, which subsequently leads to tissue damage [4]. Since there is an established correlation between inflammation and oxidative damage, the aim of this review is to present mechanisms of oxidative stress induced by COVID-19 infection and describe antioxidant activity of various treatment agents that may be useful in the therapy of novel coronavirus infection.

## 2. COVID-19 Pathophysiology and Oxidative Stress

**Figure 1:**
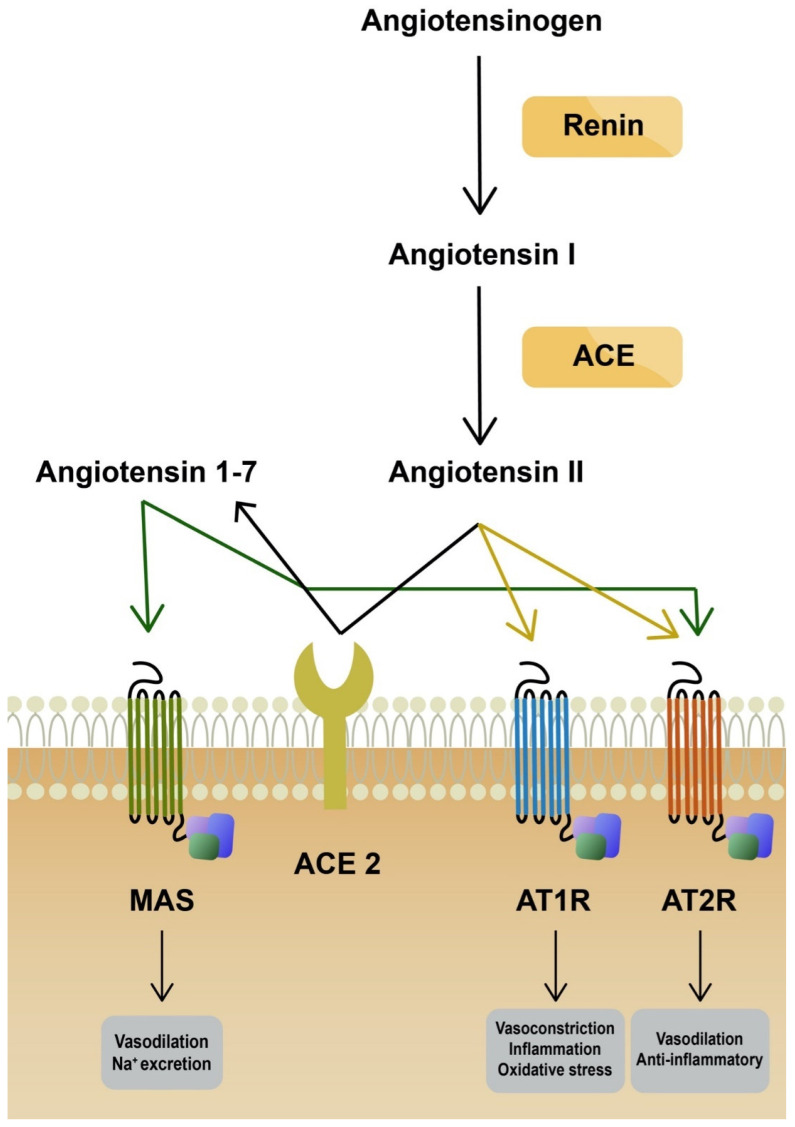
Angiotensin pathways: ACE—angiotensin convertase enzyme; ACE2—angiotensin convertase enzyme type 2; AT1R—angiotensin II type 1 receptor; AT2R—angiotensin II type 2 receptor.

**Figure 2:**
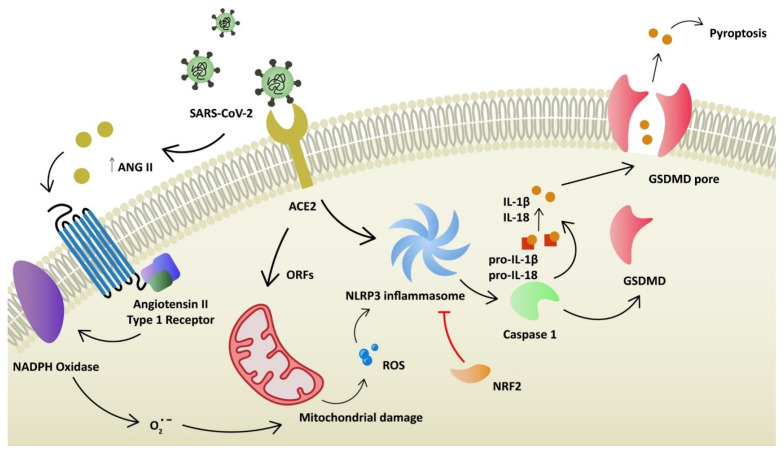
Hypothesized intracellular processes after infection of SARS-CoV-2, leading to mitochondrial damage, production of reactive oxygen species and pro-inflammatory state of the cell: ACE2—angiotensin convertase enzyme 2; ANG II—angiotensin II; ORF—open reading frame; ROS—reactive oxygen species, GSDMD—gasdermin D.

Angiotensin-converting enzyme 2 (ACE2) plays a key role as a binding domain for the spike protein of the virus and its entry receptor [5]. It is a carboxypeptidase that can regulate the renin angiotensin system (RAS). The main function of ACE2 is to transform angiotensin II (Ang II) into angiotensin 1–7, which leads to vasodilation. ACE2 protein is expressed in various tissues, including the digestive tract, kidney, heart, vasculature and eye, among others [6]. In the respiratory system, the expression of ACE2 is limited and restricted to specific cell subtypes, especially pneumocytes type II [7]. Several other receptors used by SARS-CoV-2 to invade host cells have been identified: TMPRSS2, TMPRSS4 (activation of virus proteins), GRP78, CD147 and AXL [8,9]. Expression of ACE2 is limited in the respiratory system. Since it is strongly involved in a severe course of disease, it was proven that coronavirus can upregulate ACE2 expression through interferon to enhance infection [6]. On the other hand, SARS-CoV-2 can downregulate the ACE2 receptor. Consequently, RAS is dysregulated, which leads to acute lung injury, as ACE2 protects lung cells [10]. The reduced expression of ACE2 increases the level of Ang II [11]. Ang II stimulates the secretion of aldosterone and is a potent vasoconstrictor. More importantly, it stimulates angiotensin receptor type 1 (AT1R). AT1R and AT2R are two Ang II receptors. The stimulation of these has opposing effects. AT1R promotes vasoconstriction, enhances inflammation and promotes oxidative stress (Figure 1). Ang II binding to AT1R activates NADPH oxidase, which produces superoxide anion (O_2_^−^), responsible for mitochondrial damage and further production of reactive oxygen species (ROS) [12,13]. It was already demonstrated by Papola, F. et al., that AT1R antibodies could play a protective role in SARS-CoV-2 infection [14]. On the contrary, AT2R promotes anti-inflammatory and antifibrotic mechanisms. In addition, AT2R induces vasodilation through cGMP and nitric oxide (NO) [15]. Furthermore, SARS-CoV-2 can directly compromise mitochondrial function through its open reading frames (ORFs), leading to increased levels of mitochondrial DNA and mitokine fibroblast growth factor 21 (FGF-21) [16,17]. Elevated levels of ROS are one of the stimulation factors for NOD-, LRR- and pyrin domain-containing protein 3 (NLRP3) inflammasome activation. NLRP3 belongs to a family of pattern-recognition receptors (PRRs). PRRs respond to damage-associated molecular patterns (DAMPs) and pathogen-associated molecular patterns (PAMPs) and, as a result, activate pro-caspase-1. Caspase-1 stimulates pro-interleukin-1β and pro-interleukin-18 to their mature forms (Figure 2). Consequently, regulatory processes of vasodilation, pain threshold and fever are induced. The production of interferon γ (IFNγ) is increased and adaptive immunity is regulated [18]. Furthermore, NLRP3 inflammasome activates gasdermin D (GSDMD) to form pores in the membrane, inducing a death pathway called pyroptosis [19]. It is demonstrated that novel coronavirus directly promotes the assembly of the NLRP3 inflammasome through the N-protein [20]. NLRP3 also correlates with the pathophysiology of diseases, such as atherosclerosis, gout, non-alcoholic fatty liver disease (NAFLD) and diabetes [21]. Moreover, the elevation of neutrophil extracellular traps (NET) was observed in infected patients. It was demonstrated that NETosis was induced in ROS-dependent (S2 protein) and ROS-independent pathways (N and S1 proteins) [22,23]. NETs are responsible for eliminating pathogens and preventing dissemination. However, they are also linked with tissue damage, the promotion of deep vein thrombosis and impaired wound healing [24]. ROS elevation, NLRP3 activation and NETosis are considered to take part in dysregulated immunological responses known as cytokine storms. Oxidative stress is associated with the pathogenesis of various diseases in respiratory (asthma, COPD), neurological (Alzheimer’s disease, Parkinson’s disease) and cardiovascular (atherosclerosis) systems. It has also been linked with cancer development and autoimmune diseases such as rheumatoid arthritis [25]. It is observed that SARS-CoV-2 infection upregulates oxidative stress genes, together with the elevation of oxidative stress markers in infected patients [26,27]. Additionally, the level of antioxidants and some trace elements (selenium, zinc, magnesium) were reduced in critically ill patients [28,29]. Antioxidative mechanisms (enzymatic and non-enzymatic) prevent the generation and accumulation of ROS. Reductors counteract the toxic effects of free radicals (peroxidation of membrane lipids, protein glycation or inactivation of enzymes) and restore cell homeostasis. Non-enzymatic agents (e.g., vitamin C, E, uric acid) cleave ROS and break the cascade [30]. Superoxide dismutase (SOD) and catalase are examples of enzymatic components of the antioxidant defense system [31]. An antioxidant is a substance that prevents the oxidation of potential substrates. It should chelate redox metals and remove free radicals. Ideally, such agents should positively influence gene expression and support the maintenance of physiological cell functions [32].

Severe courses of COVID-19 infection are associated with hypoxia. Therefore, oxygen therapy is a key element in patient management. Various interventions have been proposed to improve saturation, including hyperbaric oxygen therapy (HBOT), noninvasive and invasive ventilation, and extracorporeal membrane oxygenation (ECMO). In a hyperbaric chamber, patients breathe air with an oxygen concentration of almost 100%. More oxygen is dissolved in plasma in high-pressure environments. HBOT therapy was applied in COVID-19 patients with severe hypoxia and proved to be more efficient in correcting saturation compared to standard therapy [33]. Hyperoxia induced by HBOT seems to decrease pro-inflammatory cytokines through the inhibition of nuclear factor kappa B (NF-κB), which induces the expression of pro-inflammatory genes [34]. A recent study by Luo, P. et al., highlights the toxicity of high oxygen concentrations and suggests combined hydrogen–oxygen therapy. Hydrogen has the ability to diminish ROS, which could prevent hyperoxia-induced organ damage [35].

## 3. Glutathione

One of the most important antioxidant agents in human organisms is glutathione, which plays a significant role in maintaining redox homeostasis. A reduced form of glutathione (GSH) is capable of neutralizing ROS due to the activity of glutathione peroxidase, a selenium-containing enzyme. It catalyzes lipid peroxide and a hydrogen peroxide reduction. As a result, glutathione is converted into its oxidized form (GSSG) and requires glutathione reductase to regenerate GSH [36]. It is observed that SARS-CoV-2 infection leads to a reduced activity of GSH, glutathione peroxidase and reductase [28,37]. Nevertheless, the introduction of GSH is not considered as effective due to its low bioavailability and reduced potential to infiltrate cell barriers. However, GSH regeneration can be achieved using N-acetylcysteine (NAC). NAC is a L-cysteine derivative, and it has been used in the treatment of a variety of diseases, including chronic obstructive pulmonary disease, HIV infection and neurological disorders, among others. It is demonstrated that NAC has anti-inflammatory, antimicrobial and antioxidant properties [38]. After administration, it is transformed into cysteine, which is subsequently used to synthesize GSH in hepatocytes [39]. Several studies examined the clinical course of COVID-19 patients treated with NAC. According to Izquierdo JL et al. who evaluated NAC administration together with standard therapy in infected patients, the introduction of NAC resulted in the better survival of patients, but there were no differences regarding the length of hospital stays or intensive care unit (ICU) admission [40]. On the other hand, de Alencar JCG et al., performed a double-blind, randomized study comparing intravenous injections of NAC and placebo. There were no differences in case of mortality, need for mechanical ventilation or ICU admission [41]. Similar results were observed in a study by Taher, A., et al., where significant differences were not achieved between NAC and placebo groups within the aforementioned parameters [42].

Another treatment agent that is capable of regenerating glutathione is α-lipoic acid (ALA) or its reduced form, dihydrolipoic acid (DHLA). ALA plays a significant role in an organism, involved as a cofactor in the Krebs cycle, and the lipid and glucose metabolism. Additionally, it takes part in gene transcription and eliminates heavy metals [43]. Zhong, M., et al., randomized 17 COVID-19 patients into control and ALA groups. Compared with placebo, there was a decreased Sequential Organ Failure Assessment (SOFA) score, and an increased mortality rate. However, statistical significance was not achieved [44]. An in vitro study by Uberti, F., et al., evaluated the effect of ALA combined with palmitoylethanolamide (PEA) on lung cells under conditions of a stimulated cytokine storm. The authors demonstrated that an ALA and PEA combination could decrease ROS production [45]. Horowitz RI et al. reported that application of glutathione, NAC and ALA in the treatment of COVID-19-related pneumonia in two patients resulted in an improvement in clinical courses and the alleviation of dyspnea [46].

## 4. AT1R Antagonists

SARS-CoV-2 infection leads to increased levels of Ang II, which subsequently binds to AT1R, resulting in NADPH oxidase activation, which is considered to be one of the most significant sources of ROS production. Angiotensin receptor blockers (ARB), or sartans, are widely used in patients with hypertension or heart failure. However, increasing evidence suggests that ARBs could also possess antioxidant activities [47]. Telmisartan (Tel) is a long-lasting AT1R antagonist with a potency power comparable to the antihypertensive activity of other agents, such as beta blockers and ACE inhibitors [48]. In vitro and rat model studies show that Tel upregulates GSH, SOD and nuclear factor erythroid 2-related factor 2 (NRF2), together with the inhibition of NADPH oxidase [49,50]. According to Reus, P., et al., Tel reduces SARS-CoV-2 replication in cell lines expressing ACE2 [51]. Furthermore, in a randomized clinical trial by Duarte, M., et al., a twice-daily Tel dose of 80 mg was given to COVID-19 patients. In comparison to the control group (standard care), the decrease in C-reactive protein (CRP) levels was more rapid, together with shorter hospital stays, a reduced need for ICU admission and lower mortality rates [52]. Tel is further being evaluated as an anti-COVID agent in several other clinical trials (NCT04510662, NCT04359953, NCT04715763). Candesartan (Cand) represents another agent from ARBs that is considered the most efficient one in crossing the blood–brain barrier [53]. The mouse insulinoma cell line (MIN6) and mouse islets cells with induced insulin resistance were treated with Cand. It was proven that Cand led to a decreased level of ROS. In particular, it attenuated NAD(P)H oxidase activity. Moreover, Cand treatment down-regulated the expression of uncoupling protein 2 (UCP-2) mRNA, which is an anion carrier involved in the regulation of cellular homeostasis, energy production, oxidative stress and cell survival [54]. Furthermore, in rats administered with Cand, a lower level of amphetamine-induced ROS (measured as lipid and protein peroxidation) was observed [55]. Moreover, in patients diagnosed with essential hypertension, a 12-week-long Cand treatment resulted in decreased urine concentrations of 8-epi-Prostaglandin and 8-hydroxydeoxyguanosine, an index of in vivo oxidative stress and a biomarker of oxidative DNA damage, respectively [56]. It has also been demonstrated that Cand has an immunomodulatory effect on the cytokine storm, which is a hallmark of COVID-19. Elkahloun et al. proved that COVID-19 infection leads to an upregulation of 210 genes associated with the interferon pathway, cytokines and chemokines. Cand treatment decreases the expression of genes responsible for pro-inflammatory cytokines, including IL-1β, IL-6 and TNF-α [57]. Furthermore, a decreased CRP level was observed in patients with essential hypertension after the treatment with Cand; however blood pressure levels were not changed in comparison to the control group [56]. Additionally, in a prospective non-randomized open-label study by Lukito et al., it has been proven that Cand leads to shortened hospital stays in COVID-19 patients. Moreover, in the non-obese subgroup, the time to receive a negative swab and the time to see an improvement in chest X-rays were reduced [58]. Another ARB representative that is considered to play a significant role in reducing the negative consequences of the SARS-CoV-2 infection is Losartan (Lor). Similar to Cand, Lor reduces oxidative stress via the in vivo suppression of UCP-2 and NAD(P)H oxidase inhibition in β-cells [59]. In addition, lipopolysaccharide (LPS)-induced inflammatory state, described by elevated levels of IL-1β and TNF-α, was dose-dependently reduced in the mice hippocampal tissue due to the pretreatment with Lor. On the other hand, pretreatment with Lor did not increase the level of anti-inflammatory cytokine IL-10. Nevertheless, pretreatment with Lor resulted in a lower malondialdehyde concentration, which is a marker of lipid peroxidation, suggesting that it has a protective effect on hippocampal oxidative stress inductions as a result of LPS systemic injections [60]. Furthermore, Lor was applied in mice in order to counteract age-related increased oxidative stress and activated inflammatory pathways, changes associated with sarcopenia development in humans. Alterations in RAS are also involved in sarcopenia. Lor treatment resulted in a significant decrease in IL-6 serum levels as well as an increase in mRNA expression of glutathione peroxidase and catalase in quadriceps muscle in comparison to the control group. [61]. However, in a blinded, placebo-controlled randomized clinical trial Lor did not improve the health state of hospitalized COVID-19 patients. A maximal Lor dose did not reduce lung injury, which was measured with the oxygenation ratio (PaO_2_:FiO_2_) [62]. It is worth mentioning that there is also a promising study regarding the use of AT1R blockers to inhibit NLRP3 inflammasomes [63].

## 5. NRF2 Activators

**Figure 3:**
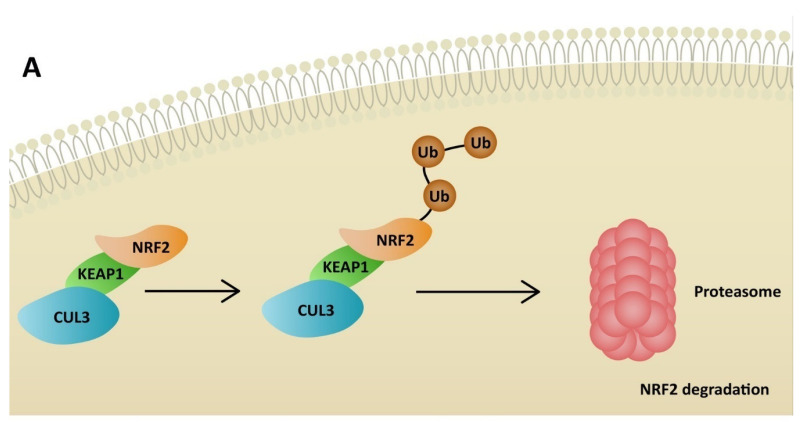

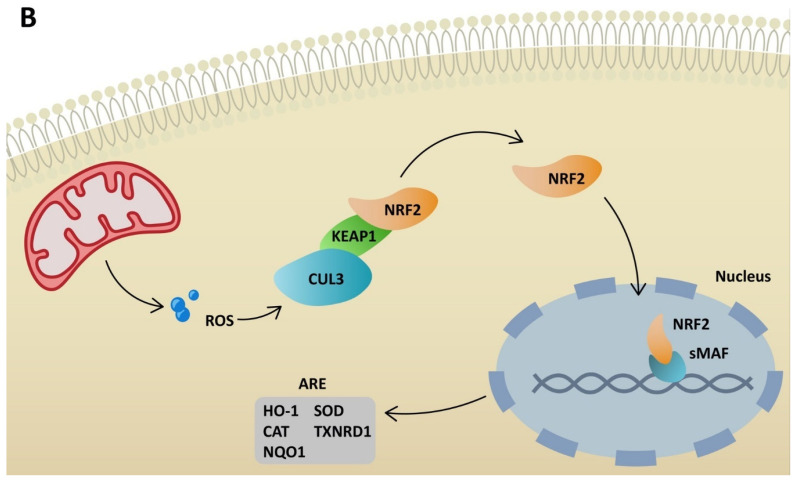
Degradation of NRF2 due to KEAP1 activity (**A**) and activation of antioxidant response elements in stressful conditions by NRF2 (**B**). ROS—reactive oxygen species; CUL3—cullin 3; KEAP1—Kelch-like ECH-associated protein; NRF2—nuclear factor erythroid 2-related factor 2; HO-1—heme oxygenase-1; CAT—catalase; NQO1—NADPH-quinone oxidoreductase-1; SOD—superoxide dismutase; TXNRD1—thioredoxin reductase 1.

**Figure 4:**
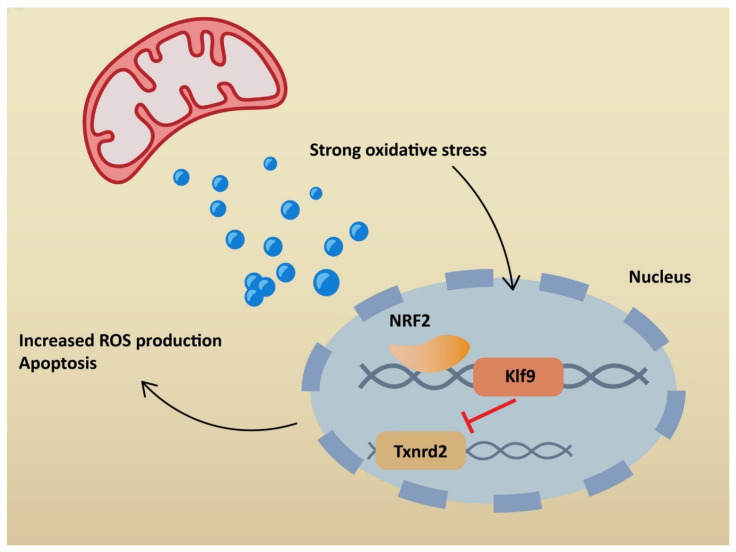
Cellular effects of high-dose ROS stimulation: ROS—reactive oxygen species; NRF2—nuclear factor erythroid 2-related factor 2; KLF9—Kruppel-like factor 9; TXNRD2—thioredoxin reductase 2.

NRF2 plays a significant role in antioxidant pathways. It activates the transcription of genes containing antioxidant response elements (ARE). In non-stressful conditions, NRF2 is constantly degraded in the proteasomal pathway due to ubiquitination by Kelch-like ECH-associated protein (KEAP1). NRF2 degradation is inhibited in stressful conditions, when KEAP1 is oxidized [64]. Therefore, NRF2 penetrates the nucleus and binds to sMAF proteins, leading to ARE activation (Figure 3). Consequently, NRF2 restores redox homeostasis, but also decreases ROS production. Many agents activate NRF2 through KEAP1 inhibition. ROS is responsible for inducing inflammation via the NLRP3 inflammasome, but at the same time activates NRF2, which restores homeostasis. Additionally, NRF2 may inhibit the transcription of genes related to NLRP3 [65]. On the other hand, cell stimulation with high-dose ROS makes NRF2 activate Kruppel-like factor 9 (*Klf9)*. *Klf9* further stimulates ROS production by repressing NRF2 target genes, such as thioredoxin reductase 2 (*Txnrd2),* with antioxidant activity (Figure 4) [66]. Gümüs, H et al. demonstrated that SARS-CoV-2 infection decreases the level of NRF2 [67]. One of the treatment agents that activates NRF2 is dimethyl fumarate (DMF), typically used to treat multiple sclerosis. The adverse effects of DMF include B and T cell depletion and lymphopenia, which could result in an increased risk of severe SARS-CoV-2 infection. However, modulating the immune system through NRF2 activation might play a protective role for infected patients. It has already been proven that DMF is capable of suppressing SARS-CoV-2 replication and inhibiting an inflammatory response to the virus [68]. Other agents that are able to activate NRF2 are flavonoids. Resveratrol (Res) is a plant derivative found in several fruits that has multiple beneficial effects, including antidiabetic, cardioprotective and antioxidant effects, among others [69]. Despite NRF2 stimulation, it has been demonstrated that Res also reduces ACE2 expression in adipocytes [70]. Additionally, according to an in vitro study by Pasquereau S et al., Res inhibits SARS-CoV-2 replication [71]. Several clinical trials are being conducted to evaluate Res as a potential anti-COVID-19 drug (NCT04400890, NCT04799743, NCT04542993). Sulforaphane (SFN) is another plant derivative found in several vegetables capable of activating NRF2. Mazarakis N et al. demonstrated that SFN can decrease the viral load of respiratory syncytial virus (RSV) in lung epithelial cells, together with activating antioxidant genes [72]. A COVID-induced cytokine storm is characterized by the extensive activity of several chemokines and interleukins. SFN can modulate cytokine storms by suppressing interleukin-6 and interleukin-8 [73]. According to Ordonez AA, SFN inhibits SARS-CoV-2 replication and works synergistically with remdesivir, a broad-spectrum antiviral drug approved by the Food and Drug Administration (FDA) for COVID-19 treatment [74]. Calcitriol, an active form of vitamin D, has the ability to activate the NRF2 signaling pathway [75,76]. According to a randomized study by Elamir YM et al., calcitriol treatment significantly improved the oxygenation index in COVID-19 patients compared to the control group [77]. Furthermore, it was demonstrated by Oristrell J et al. that calcitriol supplementation decreased the risk of COVID-19 mortality in patients with chronic kidney disease [78].

## 6. NLRP3 Antagonists

A severe course of SARS-CoV-2 infection is associated with a dysregulated immunological response. The NLRP3 inflammasome plays a significant role in this process via activating caspase-1, interleukin-1 and GSDMD. The inhibition of NLRP3 has been broadly discussed as a potential COVID-19 treatment method. Nevertheless, NLRP3 has also been linked with the pathophysiology of other diseases, such as diabetes type 2 (DM2) and its complications [79]. It has been demonstrated that the first-line DM2 agent, metformin, is capable of inhibiting the NLRP3 inflammasome independently or via activating AMP-activated protein kinase (AMPK) [80,81,82]. According to meta-analysis by Ganesh A et al., the use of metformin in DM2 and COVID-19 patients was associated with a significant decrease in mortality compared to controls [83]. In addition, it has been found that dapagliflozin (DAPA), another DM2 agent, inhibits NLRP3 inflammasome [84]. However, according to a randomized phase 3 clinical trial, DAPA treatment did not reduce mortality nor improve clinical outcomes in patients with COVID-19 [85].

Tranilast (TR) is a tryptophan derivative used since the 1980s to treat asthma. It suppresses histamine release and inhibits COX activity and interleukin secretion. Additionally, it has shown antiproliferative, anticarcinogenic and antioxidant effects [86]. According to Huang Y et al., TR directly blocks the NLRP3 complex, resulting in the reduced expression of pro-interleukin- 1β [87]. In a randomized controlled trial, TR was evaluated as an adjunctive treatment in COVID-19 patients. TR exposure decreased the activity of inflammatory cytokines and shortened the length of hospital stays. On the other hand, there were no differences in mortality and ICU admissions between patients receiving TR and the control group [88].

Fucoidan is a natural polysaccharide found in brown seaweed that is considered to have multiple beneficial therapeutic effects, including anticarcinogenic, antithrombotic and immunomodulatory effects. Fucoidan was found to have a positive impact on patients with diabetes [89]. Furthermore, it was demonstrated that fucoidan could be effective against influenza and hepatitis B viruses [90]. According to Cheng Y et al., fucoidan can inhibit the NLRP3 inflammasome via activating the p62 protein that enhances autophagy [91]. Ex vivo studies showed that fucoidan restored the mitochondrial potential of peripheral blood mononuclears from recovered COVID-19 patients [92]. Additionally, it was demonstrated that fucoidan, together with other polysaccharides from seaweed, could inhibit SARS-CoV-2 entry to the host cell [93].

## 7. Conclusions

This review summarizes the mechanisms leading to increased oxidative stress in COVID-19 infections and current evidence regarding the use of various agents with antioxidant properties. A severe course of COVID-19 is associated with a dysregulated immunological response, which could be treated with AT1R antagonists, NRF2 agonists or NLRP3 inhibitors. Neutralizing monoclonal antibodies represent new and promising therapy for COVID-19 patients. Antibodies can control virus-induced cytokine storm (tocilizumab, secukinumab) or directly target S protein and inhibit virus entry (casirivimab, imdevimab). The administration of monoclonal antibodies has been associated with reduced mortality rates and shorter hospital stays [94,95]. Some of the presented agents are known drugs used in the treatment of other diseases, such as metformin (DM2) or tranilast (asthma), while others are plant derivatives with multiple beneficial effects (resveratrol, sulforaphane). Despite their antioxidant properties, some agents possess direct antiviral effects (sulforaphane, fucoidan). More clinical trials are needed to evaluate the safety and outcomes of presented agents in monotherapy, or together with the FDA-approved antiviral drug, remdesivir.

## Data Availability

Not applicable.
